# Risk of Pneumonia Among Patients With Chronic Kidney Disease in Outpatient and Inpatient Settings: A Nationwide Population-Based Study

**DOI:** 10.1097/MD.0000000000000174

**Published:** 2014-12-12

**Authors:** Che-Yi Chou, Shu-Ming Wang, Chih-Chia Liang, Chiz-Tzung Chang, Jiung-Hsiun Liu, I-Kuan Wang, Lien-Cheng Hsiao, Chih-Hsin Muo, Chiu-Ching Huang, Ruey-Yun Wang

**Affiliations:** From the Division of Nephrology and Kidney Institute (C-YC, S-MW, C-CL, C-TC, J-HL, I-KW, C-CH), Department of Internal Medicine; College of Medicine (C-YC, S-MW, C-CL, C-TC, J-HL, I-KW, L-CH, C-HM, C-CH); Division of Cardiology (L-CH), Department of Internal Medicine; Management Office for Health Data (C-HM), and Department of Public Health (R-YW), China Medical University and Hospital, Taichung, Taiwan.

## Abstract

Patients with chronic kidney disease (CKD) are more at risk for pneumonia than the general population. Patients with pneumonia are usually treated as outpatients. However, previous studies were conducted on the basis of inpatient pneumonia. This method may underestimate the risk of pneumonia in patients with CKD. Therefore, we investigated the risk of pneumonia among CKD patients in both outpatient and inpatient settings.

A total of 15,562 patients with CKD and 62,109 individuals without CKD (matched for age and gender) were taken as subjects in the Longitudinal Health Insurance Database of Taiwan National Insurance from 1996 to 2010. The incidence density rates of inpatient and outpatient pneumonia were calculated. The risk factors associated with pneumonia were analyzed using Cox proportional hazard models with adjustments for confounders.

The incidence density rate of pneumonia was 65.6 per 1000 person-years in patients with CKD and 28.4 per 1000 person-years in individuals without CKD. The incidence density rate of inpatient pneumonia was 43.3 per 1000 person-years in patients with CKD and 16.6 per 1000 person-years in individuals without CKD. CKD was associated with increased risk of pneumonia (adjusted hazard ratio [aHR], 1.97; 95% confidence interval [CI], 1.89–2.05; *P* < 0.001), outpatient pneumonia (aHR, 1.40; 95% CI, 1.31–1.49), and inpatient pneumonia (aHR, 2.17; 95% CI, 2.07–2.29, *P* < 0.001). Patients’ comorbidities, including diabetes, cardiovascular disease (CVD), asthma, and chronic obstructive pulmonary disease (COPD), were independently associated with increased risk of pneumonia.

CKD is associated with the increased risk of both outpatient and inpatient pneumonia. This association is independent of comorbid diabetes, CVD, asthma, and COPD.

## INTRODUCTION

Pneumonia is one of the most common sources of infectious morbidities in patients with chronic kidney disease (CKD). Pneumonia in patients with CKD is associated with increased hospitalization, cardiovascular events,^1,2^ and mortality.^3,4^ The pneumonia-related mortality rate of patients with CKD is 14 to 16-fold higher than that of the general population.^4^ The risk of inpatient pneumonia and mortality within 30 days is increased with the decline of renal function in patients with CKD.^5^ However, all previous studies were based on the incidence of inpatient pneumonia. This condition may underestimate the risk of pneumonia because most treatments for pneumonia are provided in outpatient settings. This study thus aims to determine the incidence of both inpatient and outpatient pneumonia in patients with CKD using the National Health Insurance (NHI) data of Taiwan.

## MATERIALS AND METHODS

This work is a retrospective cohort study using an encrypted database from the Bureau of National Health Insurance (BNHI). The Institutional Review Board of China Medical waived the need for informed consent. The Longitudinal Health Insurance Database (LHID) of the Taiwan National Health Research Institute (NHRI) was released by BNHI. LHID included all medical records from 1996 to 2010. From these records, one million people were randomly selected. No significant differences were found in the sex and age distributions between LHID and all individuals in the NHRI. The International Classification of Disease Revision, Ninth Clinical Modification (ICD-9-CM) was used for the diagnosis codes. A 1:4 study design was used because an increase in controls did not increase statistical efficiency.^6^

### Study Sample

**FIGURE 1:**
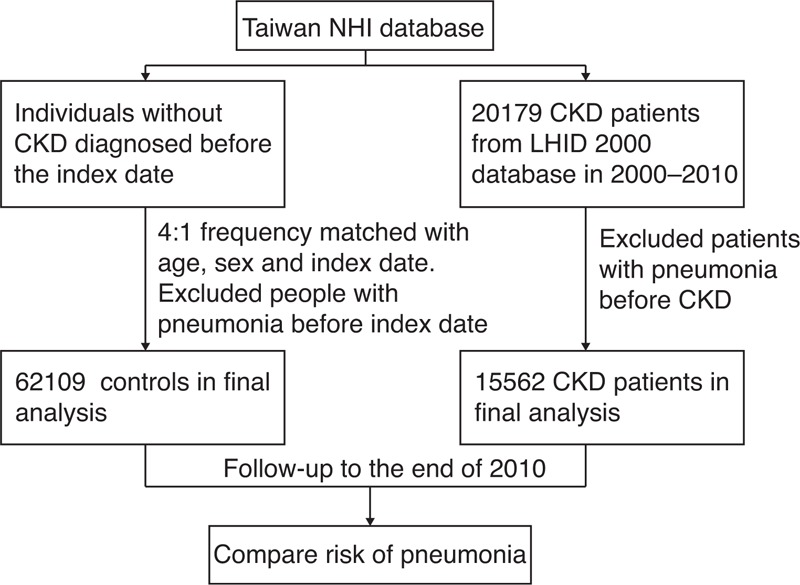
Flowchart of the study design. CKD = chronic kidney disease, LHID = Longitudinal Health Insurance Database, NHI = National Health Insurance.

Patients with CKD were defined as patients with ICD-9-CM code 585 from LHID who are not in the catastrophic illness patient database. The patients included in this database are those with CKD stages 3 to 5 without renal replacement therapy, including hemodialysis, peritoneal dialysis, and kidney transplantation (Figure 1).^7^ A total of 15,562 patients with CKD and 62,109 patients without CKD (matched for age and sex) were included. Pneumonia was identified using ICD-9-CM codes 481, 482, 485, and 486. Pneumonia-related hospitalization was also recorded. The first episode of pneumonia was recorded for patients who developed more than 1 episode. Patients with pneumonia before CKD diagnosis were excluded because we focused on the primary risk of pneumonia. Time was calculated from the index date of CKD to the date of pneumonia. Comorbidities, including diabetes (ICD-9-CM code 250), hypertension (401–405), cardiovascular disease (CVD) (410–414), asthma (493), and chronic obstructive pulmonary disease (COPD) (491, 492, 494, and 496) were defined as those that have >3 medical visits.

### Statistical Analysis

The distribution of sex, age, and comorbidities for patients with CKD and for control individuals was analyzed using χ^2^ test for categorical variables or *t* test for continuous variables. The incidence density was defined as the number of pneumonia cases divided by the total follow-up person-years (expressed as per 1000 person-years). The adjusted hazard ratio (aHR) and the 95% confidence intervals (CIs) of pneumonia risk factors were analyzed using Cox proportional hazard regression models with adjustment for sex, age, hypertension, diabetes, CVD, asthma, and COPD. The statistical significance level was set at a 2-tailed probability value <0.05. All analyses were performed using SAS software version 9.1 (SAS Institute, Cary, NC).

## RESULTS

The incidence density rate of pneumonia was 65.6 per 1000 person-years in patients with CKD and 28.4 per 1000 person-years in patients without CKD (Table 1). The incidence density rate was used because the follow-up of patients without CKD was longer than that for patients with CKD. The incidence density rate of inpatient pneumonia was 43.3 per 1000 person-years in patients with CKD and 16.6 per 1000 person-years in patients without CKD. Patients with CKD had a higher rate of diabetes (*P* < 0.001), hypertension (*P* < 0.001), CVD (*P* < 0.001), asthma (*P* < 0.001), and COPD (*P* < 0.001) than patients without CKD. Patients who developed pneumonia were older than those without pneumonia and were also more likely to have CKD, diabetes, hypertension, CVD, asthma, and COPD (Table 2). Of 5536 patients with outpatient pneumonia, 1375 (24.8%) had CKD. Of 7240 patients with inpatient pneumonia, 2661 (36.8%) had CKD. The patients with inpatient pneumonia were older than patients with outpatient pneumonia. Patients with inpatient pneumonia were more likely to have diabetes, hypertension, and CVD than patients with outpatient pneumonia. The percentage of patients with asthma was lower in patients with inpatient pneumonia than in patients with outpatient pneumonia. The prevalence of patients with COPD was not significantly different between patients with inpatient pneumonia and those with outpatient pneumonia.

**TABLE 1:**
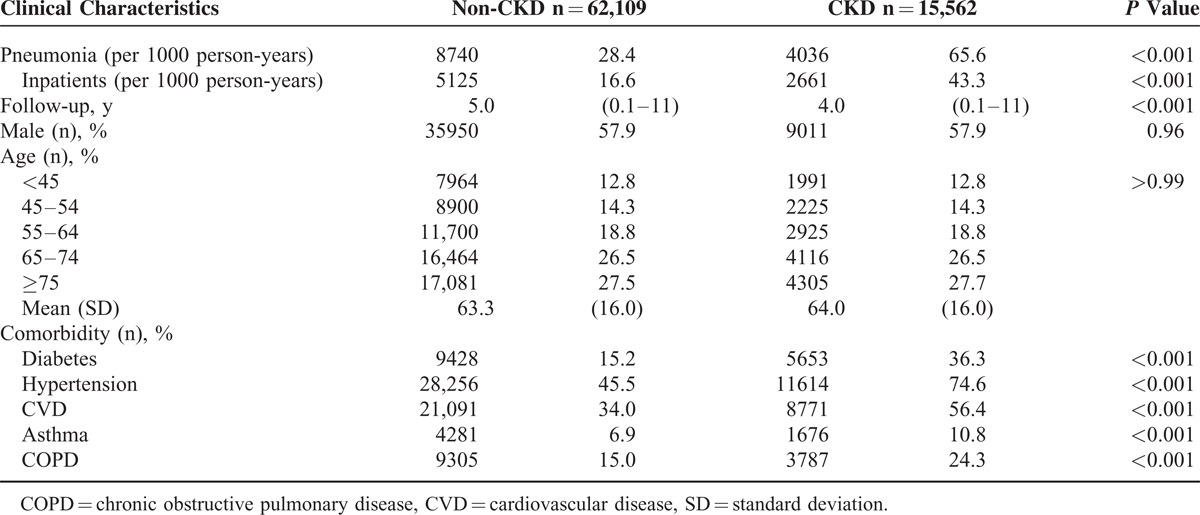
Demographic Data of Patients With and Without CKD

**TABLE 2:**
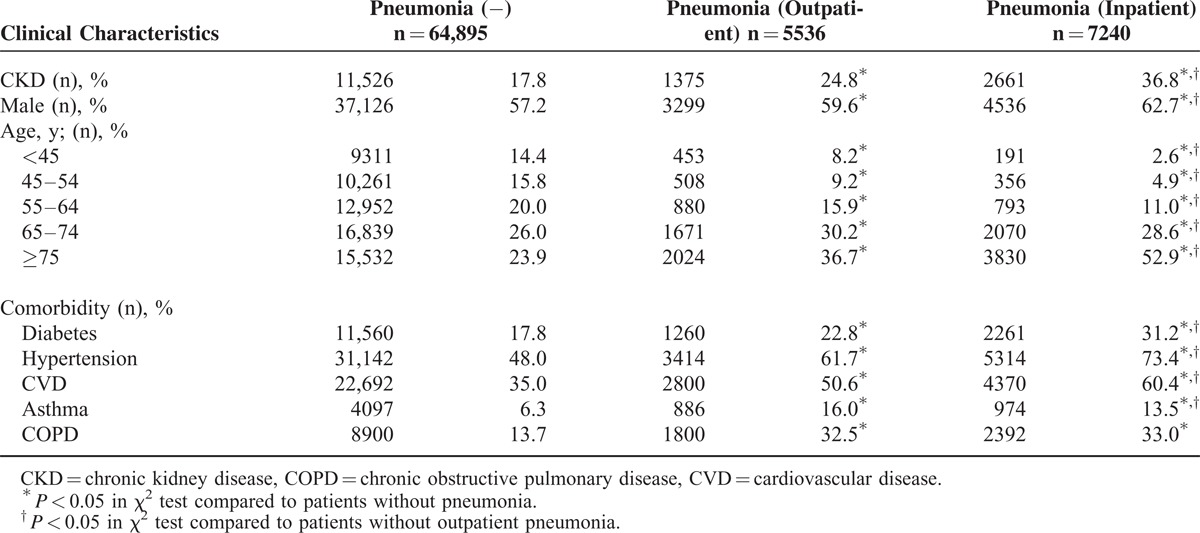
Characteristics of Patients With and Without Pneumonia

CKD, age, male gender, hypertension, diabetes, CVD, asthma, and COPD were associated with increased risk of pneumonia (Table 3). The aHR values of CKD for any type of pneumonia were 1.97 (95% CI, 1.89–2.05, *P* < 0.001) and 1.40 (95% CI, 1.31–1.49) for outpatient pneumonia and 2.17 (95% CI, 2.07–2.29) for inpatient pneumonia. Male gender (aHR, 1.13; 95% CI, 1.07–1.19), comorbid diabetes (aHR, 1.12; 95% CI, 1.05–1.19), comorbid CVD (aHR, 1.11; 95% CI, 1.04–1.20), comorbid asthma (aHR, 1.66; 95% CI, 1.56–1.79), and comorbid COPD (aHR, 1.89; 95% CI, 1.77–2.02) were independently associated with the increased pneumonia risk of outpatients. The comorbid hypertension was not associated with the increased pneumonia risk of outpatients but was associated with the increased pneumonia risk of inpatients (aHR, 1.10; 95% CI, 1.04–1.16).

**TABLE 3:**
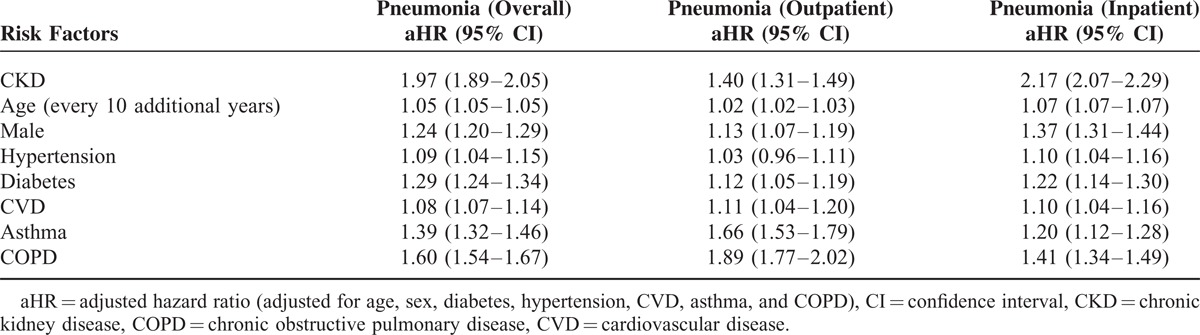
Hazard Ratio of Pneumonia Risk Factors

## DISCUSSIONS

CKD is associated with the increased risk of both outpatient and inpatient pneumonia. The risk of pneumonia was 1.97-fold higher in patients with CKD, 1.4-fold higher for outpatient pneumonia, and 2.17-fold higher for inpatient pneumonia compared with patients without CKD (Table 3). Patients with CKD have not only an increased risk of pneumonia but also an increased severity of pneumonia (indicated by hospitalization) compared with patients without CKD. The risk of overall pneumonia was higher in patients with CKD than in patients with other comorbidities (COPD, asthma, and diabetes). The risk of inpatient pneumonia was also the highest in patients with CKD. These results suggested that CKD might be an independently important contributor to the increased risk of pneumonia. The incidence of pneumonia in this study was higher than that reported in the previous studies^1,2,5,8–11^ because outpatient pneumonia was considered in the present work. The increased infection risk in patients with CKD can be explained by uremia-related impairment in monocyte,^12^ neutrophil phagocytosis,^13^ T lymphocytes,^14^ B lymphocytes,^15^ and increased cytokines.^16^ The increased risk of inpatient pneumonia in patients with CKD may be related to the comorbidity of patients with CKD because comorbidities are associated with the severity of pneumonia.^17,18^

The major strength of this study was the sample population size derived from the NHI data of Taiwan. The percentage of inpatient pneumonia was 58.6% in patients without CKD who developed pneumonia and 65.9% in patients with CKD who developed pneumonia. The high hospitalization rate of pneumonia can be attributed to 2 reasons. First, the hospitalization costs in Taiwan are low. Second, most of the hospitalization cost is covered by health insurance, and patients only need to pay 10% of the cost. The high rate of diabetes, hypertension, CVD,^3,19,20^ asthma, and COPD^21,22^ in patients with CKD was consistent with the findings of the previous studies.^21,22^

The limitations of this study are as follows. First, we could not determine the exact stage of CKD on the basis of the ICD-9-CM code 585. This condition may result in the nondifferential misclassification of CKD definition. Patients with CKD stages 1 and 2 and some patients with CKD stage 3 may be included in either patients with CKD or patients without CKD. This nondifferential misclassification may underestimate the contribution of CKD to the risk of pneumonia in the present analyses. Second, we were unable to identify CKD stages 3 to 5 exactly, and we failed to demonstrate the association of increased pneumonia risk with the worsening of CKD stages. Third, pneumonia severity index variables, such as mental status, vital signs, and laboratory data, were unavailable in LHID. Fourth, the pneumonia pathogen was associated with the severity of pneumonia^23^ but was not recorded in LHID. We failed to determine whether any specific pathogen was related to the pneumonia in patients with CKD.

In conclusion, patients with CKD have an increased risk for inpatient and outpatient pneumonia, especially pneumonia that requires hospitalization. The increased risk of pneumonia in patients with CKD is independent of their comorbidity.
